# Development and internal validation of a frailty risk stratification model for older patients with spinal tuberculosis

**DOI:** 10.3389/fmed.2026.1832932

**Published:** 2026-07-21

**Authors:** Yu Luo, Yi Wang, Bangyan Guan, Jiarong Tan, Jiahui Zhang, Jiao Luo, Ling Tang, Xin Huang

**Affiliations:** 1 Department of Nursing, Chongqing Public Health Medical Center, Chongqing, China; 2 Department of Orthopedics, Chongqing Public Health Medical Center, Chongqing, China; 3 Department of Digitalization, Chonggang General Hospital, Chongqing, China

**Keywords:** Fried frailty, nomogram, older patients, risk stratification, spinal tuberculosis

## Abstract

**Background:**

Frailty is a common geriatric syndrome in older adults and is closely associated with adverse outcomes such as prolonged hospitalization, functional decline, and mortality. Older patients with spinal tuberculosis often experience chronic inflammatory consumption, malnutrition, and activity limitation, which may contribute to a higher frailty burden. However, evidence on early identification of Fried frailty in this specific population remains limited.

**Objective:**

To develop and bootstrap internally validate a single-center model for identifying Fried frailty in older patients with spinal tuberculosis using routinely available early in-hospital clinical information.

**Methods:**

This was a single-center retrospective observational study including older patients with spinal tuberculosis who were hospitalized between January 2024 and January 2026. Variables available at admission or during the early stage of hospitalization, including demographic characteristics, nutritional and inflammatory laboratory indicators, activities of daily living, and imaging burden, were collected. Frailty status was assessed using the Fried frailty phenotype. Candidate predictors were prespecified based on the literature, clinical judgment, and early availability at admission. A multivariable logistic regression model was then used to develop the stratification model and construct a nomogram. Model performance was assessed using discrimination, calibration, and decision curve analysis, and bootstrap resampling was used for internal validation.

**Results:**

Among the 323 patients included, 260 were identified as frail, yielding a frailty prevalence of 80.50%. Multivariable analysis showed that age (OR = 1.124, 95% CI: 1.057–1.196), non-married status (OR = 6.078, 95% CI: 2.902–12.730), and the number of involved vertebrae (OR = 1.278, 95% CI: 1.014–1.610) were associated with higher odds of frailty, whereas body mass index (BMI) (OR = 0.835, 95% CI: 0.746–0.934) and Barthel Index score (OR = 0.956, 95% CI: 0.934–0.978) were associated with lower odds of frailty. The area under the receiver operating characteristic curve of the model was 0.826 (95% CI: 0.779–0.873). At the Youden-derived cutoff value of 0.835, the sensitivity was 0.665 and the specificity was 0.905. Bootstrap internal validation showed a C-index of 0.826, with an optimism-corrected C-index of 0.812. The Hosmer–Lemeshow test yielded a *p*-value of 0.215, and the Brier score was 0.125, indicating good calibration. Decision curve analysis suggested possible net benefit across a broad range of threshold probabilities; however, this exploratory finding should not be interpreted as evidence that use of the model improves clinical outcomes.

**Conclusion:**

The nomogram developed in this single-center retrospective study may serve as an auxiliary screening and early in-hospital risk-stratification tool for identifying Fried frailty in older patients with spinal tuberculosis. However, it should not be interpreted as external validation, validation of the Fried phenotype or individual clinical instruments, or evidence that use of the nomogram improves clinical outcomes. External validation and prospective impact studies are needed before broader clinical application.

## 1 Introduction

Tuberculosis (TB) remains one of the most important public health problems worldwide. According to the Global Tuberculosis Report 2025 released by the World Health Organization (WHO), an estimated 10.7 million new TB cases occurred globally in 2024, and approximately 1.23 million people died from the disease. TB remains among the top 10 causes of death worldwide, and there is still a gap between the current epidemiological situation and the WHO End TB Strategy target of a 50% reduction in TB incidence by 2025 (1). Spinal tuberculosis is a common form of secondary extrapulmonary TB and represents the most frequently affected site in osteoarticular tuberculosis. Systematic reviews have suggested that spinal tuberculosis accounts for nearly half of all osteoarticular TB cases, approximately 40–50% (2, 3). Spinal tuberculosis may lead to a series of clinical problems, including neurological impairment, spinal deformity, chronic pain, and functional limitation, thereby imposing a substantial disease burden on patients.

With the accelerating process of population aging, the risks of TB infection and adverse outcomes are increasing among older adults. Due to immune senescence, a higher burden of chronic comorbidities, and poor nutritional status, older adults are more susceptible to infection and are more likely to experience unfavorable disease progression and outcomes. Among older patients with TB, particularly those with spinal tuberculosis, frailty may be more prevalent and may have an important impact on disease progression, treatment tolerance, and long-term prognosis. However, systematic research on frailty in this population remains limited. China is one of the high-TB-burden countries worldwide and plays an important role in global TB control. It has been estimated that China accounts for approximately 6–7% of newly diagnosed TB cases worldwide (4). Although considerable progress has been made in TB prevention and control in China in recent years, the clinical characteristics and prognostic issues of older patients with spinal tuberculosis remain important concerns in both public health and clinical practice in the context of population aging.

Frailty is a common geriatric syndrome characterized by decreased physiological reserve, reduced stress resilience, and functional decline. It has been shown to be closely associated with adverse outcomes, including prolonged hospital stay, functional loss, readmission, and increased mortality (5, 6). Previous studies have reported that the prevalence of frailty is approximately 10% in community-dwelling older adults (7), whereas it may increase to 40% among hospitalized older patients and those with multiple chronic conditions (8). Importantly, early screening and timely intervention targeting modifiable risk factors may help prevent frailty progression and even reverse frailty status in some individuals (9).

Although frailty-related research has increased in recent years, existing evidence has mainly focused on general hospitalized older populations or patients with cardiovascular disease or postoperative conditions, and most frailty prediction tools have been developed in the form of nomograms (9–11). In the field of infectious diseases, emerging evidence suggests that TB is closely associated with geriatric syndromes such as frailty, sarcopenia, malnutrition, and functional decline, which may further affect treatment effectiveness and clinical outcomes (12, 13). Among hospitalized older patients with pulmonary TB, frailty is highly prevalent and has been associated with lower body mass index, polypharmacy, and functional limitation (14). For spinal tuberculosis, however, previous studies have mainly focused on perioperative risk assessment, imaging severity, and the relationship between nutritional indicators and prognosis. Some studies have attempted to introduce frailty scores into perioperative management among older patients undergoing surgery for spinal tuberculosis, but these investigations have largely been limited to surgical populations (15). Other studies have identified factors associated with postoperative mortality in older patients with spinal tuberculosis (16), and the geriatric nutritional risk index has also been reported to predict perioperative complications and long-term outcomes in this population (17).

Overall, research on the prevalence of frailty, predictors associated with frailty, and frailty stratification models in older patients with spinal tuberculosis remains limited. Frailty, as an important geriatric syndrome, has not received sufficient attention in this population, and individualized risk assessment tools integrating routinely available clinical information, nutrition-related indicators, functional status, and imaging burden are still lacking. In the context of population aging and the continuing burden of tuberculosis, early frailty stratification may help optimize inpatient geriatric tuberculosis care pathways, multidisciplinary resource allocation, and timely supportive management. Therefore, this study aimed to integrate multidimensional clinically accessible information to develop and internally validate an early frailty risk stratification model for older patients with spinal tuberculosis, with the goal of supporting early identification of high-risk individuals and in-hospital risk stratification at admission.

## 2 Materials and methods

### 2.1 Study design and participants

#### 2.1.1 Study design

This was a single-center retrospective observational study aimed at developing and internally validating an early frailty risk stratification model for older patients with spinal tuberculosis based on historical inpatient clinical data. The study population consisted of older patients with spinal tuberculosis who were hospitalized at a tertiary infectious disease specialty hospital in Chongqing, China, between January 2024 and January 2026.

#### 2.1.2 Study population and eligibility criteria

The inclusion criteria were as follows: (1) meeting the diagnostic criteria for spinal tuberculosis according to the Chinese Guidelines for the Surgical Treatment of Spinal Tuberculosis (2022 edition) (18); and (2) age ≥ 60 years, consistent with the geriatric medicine standard. The exclusion criteria were as follows: (1) concomitant severe malignant tumors or other end-stage diseases; (2) a history of severe spinal deformity or previous spinal surgery; and (3) severely incomplete clinical data.

### 2.2 Outcome definition

Frailty was assessed using the Fried frailty phenotype. Patients meeting ≥ 3 criteria were classified as frail, those meeting 1–2 criteria were classified as pre-frail, and those meeting 0 criteria were classified as non-frail. Given that this study aimed to develop an early frailty risk stratification model for binary risk identification at admission and to improve the clinical applicability of risk stratification, pre-frail and non-frail patients were combined into the non-frail group (coded as 0), while frail patients were coded as 1. For descriptive purposes, the numbers and proportions of robust, pre-frail, and frail patients were also reported separately. For the primary model development, pre-frail and robust patients were combined as the reference group because the main objective was to identify patients who met the Fried frailty threshold at admission.

### 2.3 Data sources and candidate predictors

#### 2.3.1 Data sources

Data were obtained from the hospital electronic medical record system and the Yihui nursing information system. Specifically, demographic characteristics, disease-related information, comorbidities, laboratory test results, and imaging parameters were extracted from the electronic medical record system, whereas nutritional risk scores and functional status data were obtained from the nursing information system. The Fried frailty phenotype, GDS-15 score, and SSRS score were derived from routine inpatient assessment records. A total of 340 patient records were initially retrieved. After excluding 17 cases with missing key data or incomplete information, 323 patients were ultimately included in the analysis.

#### 2.3.2 Candidate predictors

Based on the literature, clinical judgment, and the availability of variables at admission or during the early stage of hospitalization, the following candidate predictors were prespecified: demographic characteristics [sex, age, marital status, and body mass index (BMI)]; disease-related information (disease duration); comorbidities (diabetes, hypertension, and coronary heart disease); laboratory indicators (C-reactive protein, erythrocyte sedimentation rate, hemoglobin, and albumin); nutritional risk (NRS2002 score); functional status (Barthel Index score); psychological status (GDS-15 score); social support (SSRS score); and imaging parameters (anatomical distribution of lesions, number of involved vertebrae, degree of vertebral destruction, and presence or absence of paravertebral abscess).

### 2.4 Study instruments and measurement of key variables

#### 2.4.1 Frailty assessment

Frailty was assessed using the Fried frailty phenotype, which includes five components: unintentional weight loss, exhaustion, weak grip strength, slow walking speed, and low physical activity. One point was assigned for each positive component, yielding a total score ranging from 0 to 5. Unintentional weight loss was determined based on patient or family recall in combination with medical records or previous health examination records to assess body weight change over the preceding 12 months; a loss of ≥ 4.5 kg (10 lb) or ≥ 5% of body weight was considered positive. Exhaustion was assessed following the original Fried approach using standardized questions regarding the frequency of fatigue during the previous week; either related item being reported as occurring often (3–4 days/week) or most of the time (5–7 days/week) was considered positive.

Grip strength was measured using a hand dynamometer on the dominant hand. Measurements were performed 2–3 times in a standardized posture, and the maximum value was recorded. Walking speed was assessed using a 4-m straight-line walking test on a flat surface, including acceleration and deceleration segments; two measurements were obtained, and the best performance was used. Reduced grip strength was defined according to the sex- and BMI-stratified cutoff values in the original Fried criteria, and slow walking speed was defined according to sex- and height-stratified cutoff values. Low physical activity was determined by converting physical activity-related assessment items into weekly energy expenditure and applying the original Fried thresholds, namely < 383 kcal/week for men and < 270 kcal/week for women.

These assessments were preferentially completed within 24–48 h after admission. If completion within this time window was not feasible because of disease severity, pain, or necessary diagnostic procedures, the assessment was completed no later than 1 week after admission. To reflect frailty status in the early stage of hospitalization as accurately as possible, all predictors included in the model were restricted to information available at admission or during the early stage of hospitalization.

#### 2.4.2 Psychological status and social support assessment

(1) Geriatric Depression Scale (GDS)

Depressive symptoms were assessed using the 15-item Geriatric Depression Scale (GDS-15). The GDS-15 was derived by Sheikh and Yesavage from the original 30-item GDS and uses dichotomous “yes/no” responses. According to the scoring rules of the scale, responses indicative of depression are scored as 1, resulting in a total score ranging from 0 to 15, with higher scores indicating more severe depressive symptoms. The GDS-15 is commonly interpreted as follows: 0–5 points indicate a normal range, and > 5 points suggests possible depressive symptoms. The Chinese version of the GDS-15 has been reported to have a Cronbach’s α coefficient of 0.745 in older Chinese populations, indicating good reliability and validity (19).

(2) Social Support Rating Scale (SSRS)

Social support was assessed using the Social Support Rating Scale (SSRS), which includes three dimensions: subjective support, objective support, and utilization of support, comprising a total of 10 items. The total score ranges from 12 to 66, with higher scores indicating a higher level of social support. Previous studies have reported that the Cronbach’s α coefficient of this scale ranges from 0.707 to 0.741 (20).

#### 2.4.3 Nutritional risk assessment

Nutritional risk was assessed using the Nutritional Risk Screening 2002 (NRS2002). This tool mainly includes three domains: impaired nutritional status, disease severity, and age. The total score ranges from 0 to 7, with higher scores indicating greater nutritional risk. A total score of ≥ 3 is generally considered indicative of nutritional risk. In this study, the NRS2002 score was extracted from routine nursing assessment records after admission, and the first assessment performed at admission or during the early stage of hospitalization was used for analysis.

#### 2.4.4 Assessment of activities of daily living

Functional status was assessed using the Barthel Index. The Barthel Index is primarily used to evaluate activities of daily living and includes items such as feeding, bathing, dressing, toileting, bed-chair transfer, and walking. The total score ranges from 0 to 100, with higher scores indicating better functional independence in daily living. In this study, the Barthel Index was obtained from routine nursing assessment records after admission, and the first assessment performed at admission or during the early stage of hospitalization was used for analysis.

#### 2.4.5 Determination of imaging indicators

All participants underwent spinal MRI or CT at admission. Imaging data were independently reviewed by two radiologists with more than 10 years of experience who were blinded to frailty status and other clinical information. The assessment included the anatomical distribution of lesions, number of involved vertebral segments, presence or absence of paravertebral abscess, and degree of vertebral destruction. In cases of disagreement, a third senior radiologist was consulted and a consensus was reached through discussion.

##### 2.4.5.1 Classification of lesion location

According to the involved anatomical region, lesions were classified as cervical type (involving only C1–C7), thoracic type (involving only T1–T12), lumbar type (involving only L1–L5), lumbosacral/sacral type (involving S1–S5 or the L5/S1 junction), and multiregional type (involving ≥ 2 anatomical regions, such as thoracic + lumbar or thoracic/lumbar + sacral).

##### 2.4.5.2 Determination of paravertebral abscess

The presence of paravertebral abscess was determined by direct image review by radiologists; when imaging findings were atypical, the radiology report was used as an auxiliary reference for adjudication. Reports explicitly describing abscess, cold abscess, fluid collection, abscess cavity, or dynamic changes in an abscess (e.g., reduction or enlargement compared with previous imaging) were defined as indicating the presence of abscess. Reports explicitly stating no abscess, no cold abscess, or no fluid collection, or describing only nonspecific inflammatory manifestations such as soft tissue edema, swelling, or thickening without specifically mentioning abscess, were classified as indicating no abscess.

##### 2.4.5.3 Determination of vertebral destruction severity

The severity of vertebral destruction was graded according to imaging findings. With reference to previous imaging-based classification methods for spinal tuberculosis (21), lesion severity was categorized into three types. Type I indicated lesions confined to the vertebral body without vertebral collapse, with or without abscess formation and involvement of one to two intervertebral discs, and without neurological impairment. Type II indicated vertebral collapse or pathological fracture, often accompanied by destruction of one to two intervertebral discs, abscess formation, kyphotic deformity, and spinal instability, but without neurological impairment. Type III indicated severe vertebral collapse, characterized by marked kyphotic deformity and spinal instability, accompanied by neurological impairment.

### 2.5 Sample size calculation

The sample size of this study was estimated according to the principle that at least 10 outcome events are required per variable (events per variable, EPV) to improve model stability and reduce the risk of overfitting. Based on the EPV principle, the required sample size was approximately calculated using the formula: N ≥ (10 × k)/P, where k represents the number of variables planned for inclusion in the multivariable logistic regression analysis and P represents the event rate of the outcome. Based on previous literature, clinical judgment, and the main predictors intended for inclusion in the initial modeling process, 20 predictors were considered in this study. According to preliminary statistics from similar cases treated at our center, the prevalence of frailty was approximately 80% (*P* = 0.80). On this basis, the minimum required sample size was estimated to be 250 patients. Considering the potential for missing key medical record data and incomplete imaging information, an additional 10–20% of invalid samples was allowed for, resulting in a minimum required sample size of 275 patients. Ultimately, after excluding cases with missing key data or incomplete information, 323 patients were included, which met the preliminary EPV-based sample size estimate.

### 2.6 Missing data handling

A total of 340 patient records were initially retrieved in this study. After excluding 17 cases with missing key data or incomplete information that could not be used for model development, 323 patients were ultimately included in the analysis. Among these 323 patients, a small proportion of non-outcome variables still had missing values, with the missing proportions for C-reactive protein and erythrocyte sedimentation rate both being < 10%. Given the relatively low proportion of missing data, multiple imputation was used to handle missing values in order to reduce the potential bias caused by directly excluding incomplete cases. The results obtained from the imputed datasets were further compared with those from the complete-case analysis, and the findings were generally consistent, indicating that the method used for missing data handling did not substantially affect the main study conclusions and that the model showed good stability.

### 2.7 Statistical analysis

All statistical analyses were performed using R version 4.5.2, mainly with the broom, rms, pROC, rmda, car, Resource Selection, ggplot2, and mice packages for analysis and visualization. Continuous variables were described as mean ± standard deviation or median (interquartile range), depending on their distribution, while categorical variables were presented as frequencies and percentages. For between-group comparisons, continuous variables were analyzed using the independent-samples *t*-test or Mann–Whitney U test, and categorical variables were compared using the χ^2^ test or Fisher’s exact test. Candidate predictors were prespecified based on the literature, clinical judgment, and their availability at admission or during the early stage of hospitalization. Univariate analysis was first conducted to describe the associations between candidate predictors and frailty status. Variables with statistical significance in the univariate analysis (*P* < 0.05) were considered as preliminary candidates for multivariable logistic regression analysis. In addition, clinical interpretability, measurement reliability, variable availability, and multicollinearity diagnostics were considered when determining the final variables to be retained in the model. A nomogram was subsequently constructed based on the final model. Model discrimination was evaluated using the area under the receiver operating characteristic curve (AUC) and the concordance index (C-index). Model calibration was assessed using calibration curves, the Hosmer–Lemeshow goodness-of-fit test, and the Brier score. The potential net benefit of the model was explored using decision curve analysis (DCA). Internal validation was performed using bootstrap resampling (*B* = 500), and the optimism-corrected C-index was reported to assess model stability. All statistical tests were two-sided, and *P* < 0.05 was considered statistically significant.

### 2.8 Ethical approval

This study was approved by the Research Ethics Committee of Chongqing Public Health Medical Center (Approval No. 2026-018-02-KY). All study procedures were conducted in accordance with relevant guidelines and regulations. As this was a retrospective study based on electronic medical records and routine admission assessment data and did not involve any additional intervention for patients, the ethics committee approved a waiver of informed consent.

## 3 Results

### 3.1 General characteristics of the study population and frailty status

Among all included patients, 260 were identified as frail, yielding a frailty prevalence of 80.50%. According to the original Fried frailty categories, 20 patients (6.2%) were classified as robust, 43 patients (13.3%) as pre-frail, and 260 patients (80.5%) as frail. The relatively low proportions of robust and pre-frail patients suggest that this cohort represented a frailty-enriched inpatient population, likely reflecting the combined burden of advanced age, chronic spinal tuberculosis, pain, nutritional depletion, restricted ambulation, and functional limitation.

### 3.2 Univariate analysis of frailty in older patients with spinal tuberculosis

Using frailty status as the grouping variable, univariate analyses were performed for candidate variables, including demographic characteristics, nutritional status, inflammatory indicators, lesion-related characteristics, and psychosocial factors. The results showed that the Barthel Index, NRS2002 score, age, marital status, number of involved vertebrae, erythrocyte sedimentation rate (ESR), C-reactive protein (CRP), disease duration, and BMI differed significantly between the frail and non-frail groups (all *P* < 0.05). However, disease duration was not retained in the final model because it was mainly based on patient or family recall before admission and may be affected by recall bias and imprecise estimation of symptom onset. Detailed results are presented in Tables 1A,B.

**TABLE 1A:** Continuous baseline characteristics according to frailty status in older patients with spinal tuberculosis.

Variable	Number of cases (*n* = 323)	Non-frailty group (*n* = 63)	Frailty group (*n* = 260)	Statistic	*p*-value
Barthel index, points	55.09 ± 18.47	64.84 ± 15.11	52.73 ± 18.46	−5.453	< 0.001
NRS2002 score, points	2.16 ± 0.99	1.71 ± 0.73	2.27 ± 1.02	4.955	< 0.001
Age, years	68.86 ± 7.32	65.57 ± 3.81	69.66 ± 7.73	6.026	< 0.001
Number of involved vertebrae, segments	3.39 ± 1.91	2.73 ± 1.19	3.55 ± 2.01	4.213	< 0.001
ESR, mm/h	35.00 (21.00,65.00)	32.00 (16.00,52.00)	35.50 (22.00,74.00)	9,512.00	0.047
CRP, mg/L	21.00 (11.15,41.73)	19.50 (4.17,24.67)	21.58 (12.30,41.77)	9,649.00	0.028
Disease duration, days	28.00 (12.00,42.00)	30.00 (28.00,49.00)	22.00 (10.00,35.75)	6,353.00	0.006
Hemoglobin, g/L	115.91 ± 19.93	117.48 ± 17.45	115.53 ± 20.50	−0.768	0.444
SSRS score, points	35.58 ± 6.58	35.44 ± 7.64	35.62 ± 6.31	0.165	0.870
Albumin, g/L	34.92 ± 7.53	34.38 ± 4.03	35.06 ± 8.15	0.942	0.347
GDS-15 score, points	6.09 ± 3.19	5.83 ± 3.22	6.15 ± 3.18	0.728	0.468
BMI, kg/m^2^	21.24 ± 2.97	22.27 ± 2.93	20.98 ± 2.93	−3.114	0.002

Missing values before imputation were observed for CRP and ESR, with missing proportions of < 10% for both variables. Multiple imputation was used for model development, and complete-case analysis yielded generally consistent findings.

**TABLE 1B:** Categorical baseline characteristics according to frailty status in older patients with spinal tuberculosis.

Variable	Number of cases (*n* = 323)	Non-frailty group (*n* = 63)	Frailty group (*n* = 260)	Statistic	*p*-value
Marital status				15.112	< 0.001
Married	121 (37.5%)	37 (58.7%)	84 (32.3%)		
Not married	202 (62.5%)	26 (41.3%)	176 (67.7%)		
Paravertebral abscess				3.547	0.060
No	267 (82.7%)	47 (74.6%)	220 (84.6%)		
Yes	56 (17.3%)	16 (25.4%)	40 (15.4%)		
Sex				1.349	0.245
Male	180 (55.7%)	31 (49.2%)	149 (57.3%)		
Female	143 (44.3%)	32 (50.8%)	111 (42.7%)		
Hypertension				3.082	0.079
No	243 (75.2%)	42 (66.7%)	201 (77.3%)		
Yes	80 (24.8%)	21 (33.3%)	59 (22.7%)		
Vertebral destruction severity				1.173	0.556
Type I	282 (87.3%)	56 (88.9%)	226 (86.9%)		
Type II	30 (9.3%)	4 (6.3%)	26 (10.0%)		
Type III	11 (3.4%)	3 (4.8%)	8 (3.1%)		
Diabetes				0.064	0.800
No	255 (78.9%)	49 (77.8%)	206 (79.2%)		
Yes	68 (21.1%)	14 (22.2%)	54 (20.8%)		
Coronary heart disease				0.415	0.519
No	314 (97.2%)	62 (98.4%)	252 (96.9%)		
Yes	9 (2.8%)	1 (1.6%)	8 (3.1%)		
Spine region type				8.647	0.071
Cervical	9 (2.8%)	0	9 (3.5%)		
Thoracic	138 (42.7%)	30 (47.6%)	108 (41.5%)		
Lumbar	78 (24.1%)	21 (33.3%)	57 (21.9%)		
Lumbosacral/Sacral	50 (15.5%)	6 (9.5%)	44 (16.9%)		
Multiregional	48 (14.9%)	6 (9.5%)	42 (16.2%)		

### 3.3 Logistic regression analysis of frailty risk in older patients with spinal tuberculosis

Frailty status was used as the dependent variable. Variables showing statistically significant differences in the univariate analysis were considered as preliminary candidates for multivariable logistic regression analysis, together with clinical interpretability, measurement reliability, early in-hospital availability, and multicollinearity diagnostics. The coding of the independent variables is presented in Table 2. Based on clinical interpretability, variable availability, and multicollinearity diagnostics, the final model included five variables: age, marital status, BMI, number of involved vertebrae, and Barthel Index. Although disease duration showed a statistically significant difference in the univariate analysis, it was not retained in the final model because it was mainly based on patient or family recall before admission and may therefore be affected by recall bias and imprecise estimation of symptom onset. The results showed that age, non-married status, and number of involved vertebrae were associated with higher odds of frailty in older patients with spinal tuberculosis, whereas BMI and Barthel Index were associated with lower odds of frailty. For clinical interpretability, the OR for a 10-point increase in the Barthel Index was further calculated as 0.635 (95% CI: 0.503–0.801), indicating that a 10-point higher Barthel Index score was associated with lower odds of frailty. Collinearity diagnostics showed that the variance inflation factors (VIFs) for the variables in the final model ranged from 1.050 to 1.373, indicating no apparent multicollinearity. The detailed results are shown in Table 3.

**TABLE 2:** Coding of preliminary candidate variables for multivariable logistic regression analysis.

Variable	Assignment
Marital status	Married = 1; Not married = 2
Barthel Index	Entered as a continuous variable
NRS2002	Entered as a continuous variable
Age	Entered as a continuous variable
Number of involved vertebrae	Entered as a continuous variable
ESR	Entered as a continuous variable
CRP	Entered as a continuous variable
BMI	Entered as a continuous variable
Disease duration	Entered as a continuous variable

**TABLE 3:** Logistic regression analysis of frailty risk in older patients with spinal tuberculosis.

Variable	β	SE	Wald	*p*	OR	95%CI	VIF
Age	0.117	0.032	13.731	< 0.001	1.124	1.057–1.196	1.078
Marital status	1.805	0.377	22.891	< 0.001	6.078	2.902–12.730	1.373
BMI	−0.181	0.057	9.868	0.002	0.835	0.746–0.934	1.097
Number of involved vertebrae	0.245	0.118	4.330	0.037	1.278	1.014–1.610	1.050
Barthel Index	−0.045	0.012	14.703	< 0.001	0.956	0.934–0.978	1.287
Constant	−1.535	2.379	0.417	0.519	0.215	–	–

VIF, variance inflation factor, used to assess multicollinearity; a VIF < 5 suggests no apparent multicollinearity. For continuous variables, ORs are presented per 1-unit increase unless otherwise specified: age per year, BMI per kg/m^2^, number of involved vertebrae per segment, and Barthel Index per point. The OR for marital status refers to not married versus married. For clinical interpretability, a 10-point increase in the Barthel Index corresponded to an OR of 0.635 (95% CI: 0.503–0.801), calculated from the original per-point OR.

### 3.4 Development of the frailty risk stratification model for older patients with spinal tuberculosis

**FIGURE 1:**
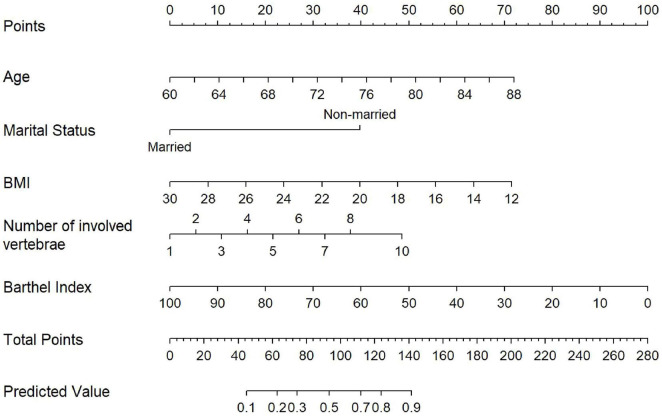
Nomogram for frailty risk stratification in older patients with spinal tuberculosis.

Based on the predictors identified in the multivariable logistic regression analysis, a frailty risk stratification model for older patients with spinal tuberculosis was constructed as follows: logit(p) = ln[P/(1 − P)] = −1.535 + 0.117 × Age + 1.805 × Marital status −0.181 × BMI + 0.245 × Number of involved vertebrae − 0.045 × Barthel Index. A nomogram was generated using R version 4.5.2 to provide a visual presentation of the stratification model (Figure 1). Scores were assigned according to the regression coefficients of each predictor. By aligning each variable with the corresponding point scale, the individual score for each factor could be obtained. The total score was calculated as the sum of the scores for all factors and was then converted into the predicted probability of frailty according to the corresponding functional relationship.

### 3.5 Evaluation and validation of the frailty risk stratification model for older patients with spinal tuberculosis

**FIGURE 2:**
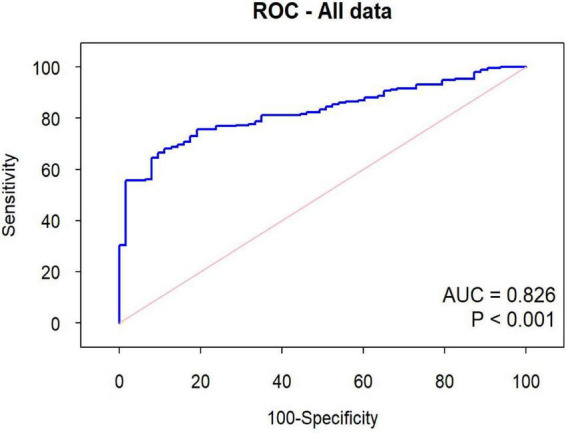
Receiver operating characteristic (ROC) curve of the frailty stratification model.

**FIGURE 3:**
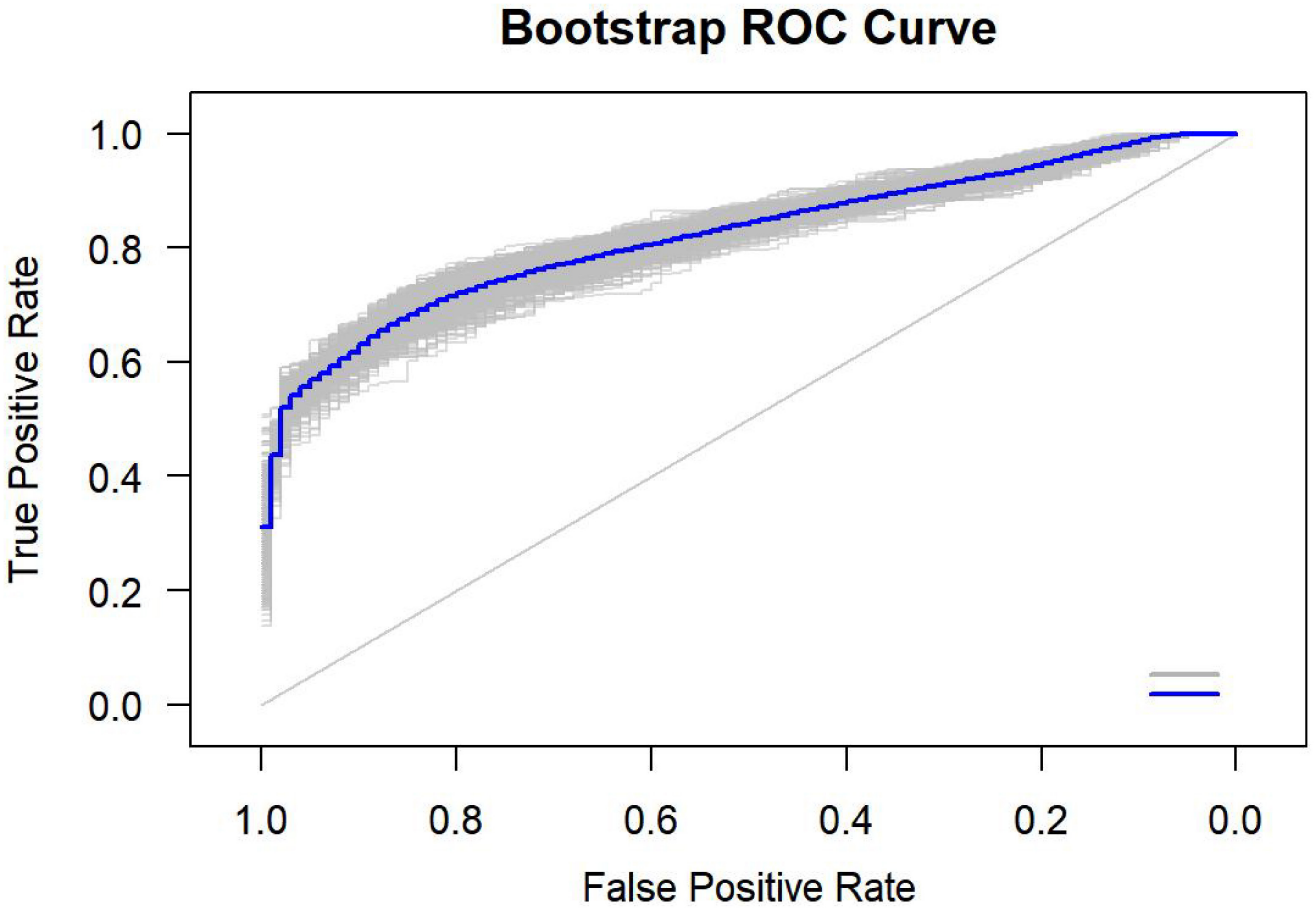
Internal validation of the frailty stratification model using bootstrap resampling.

The receiver operating characteristic (ROC) curve of the model is shown in Figure 2. The results showed that the area under the curve (AUC) was 0.826 (95% CI: 0.779–0.873), indicating good discrimination. The maximum Youden index was 0.570, corresponding to a Youden-derived probability cutoff value of 0.835. At this cutoff, the sensitivity and specificity of the model for identifying frailty were 0.665 and 0.905, respectively. Because this threshold had relatively high specificity but only moderate sensitivity, it should not be interpreted as the single optimal threshold for broad frailty screening. Clinically oriented threshold analyses are presented in section 3.7. Bootstrap internal validation (*B* = 500) showed that the original C-index of the model was 0.826 and the optimism-corrected C-index was 0.812, indicating that the model retained good discriminative ability after internal validation (Figure 3).

**FIGURE 4:**
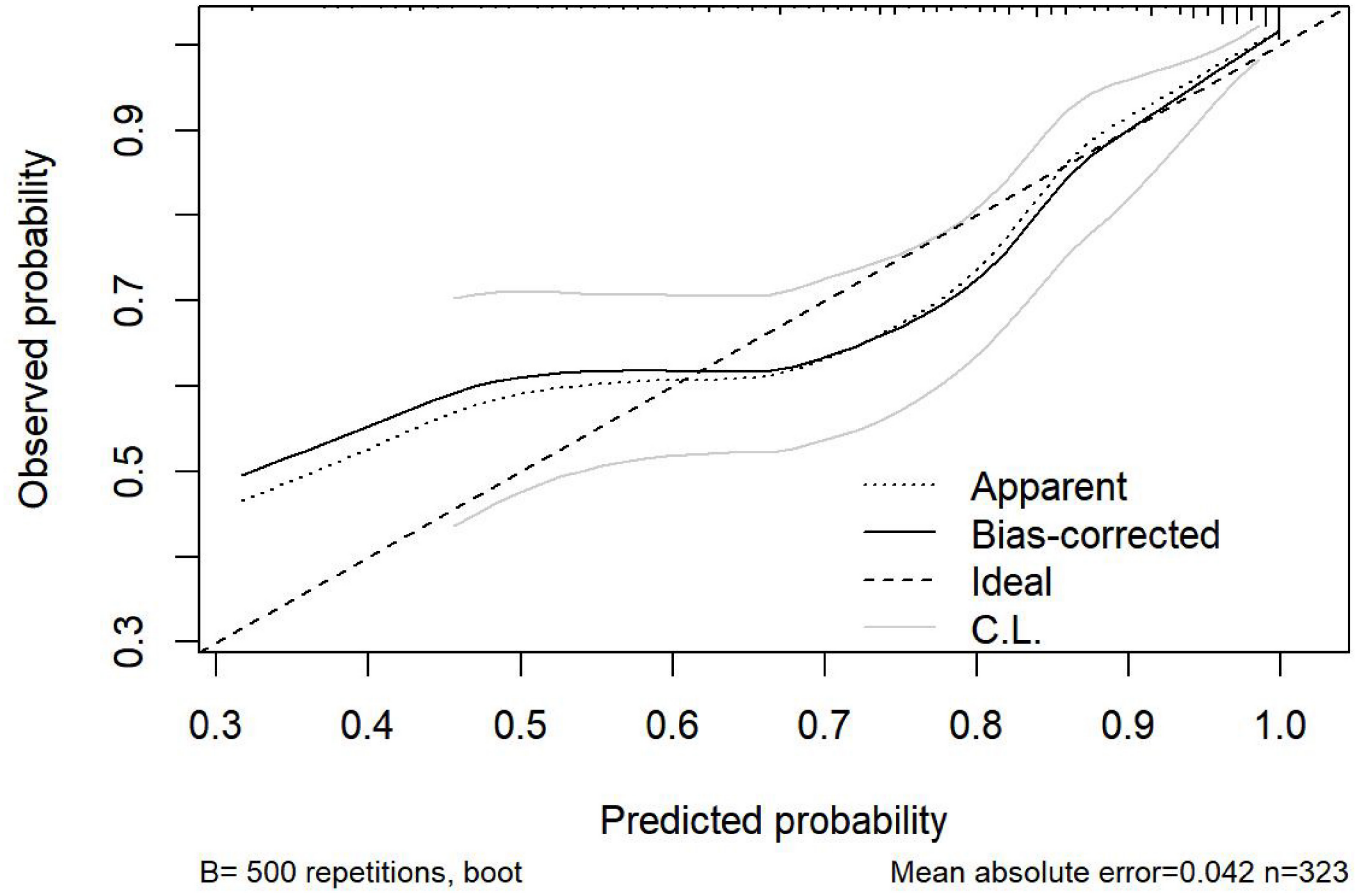
Calibration curve of the frailty stratification model.

**FIGURE 5:**
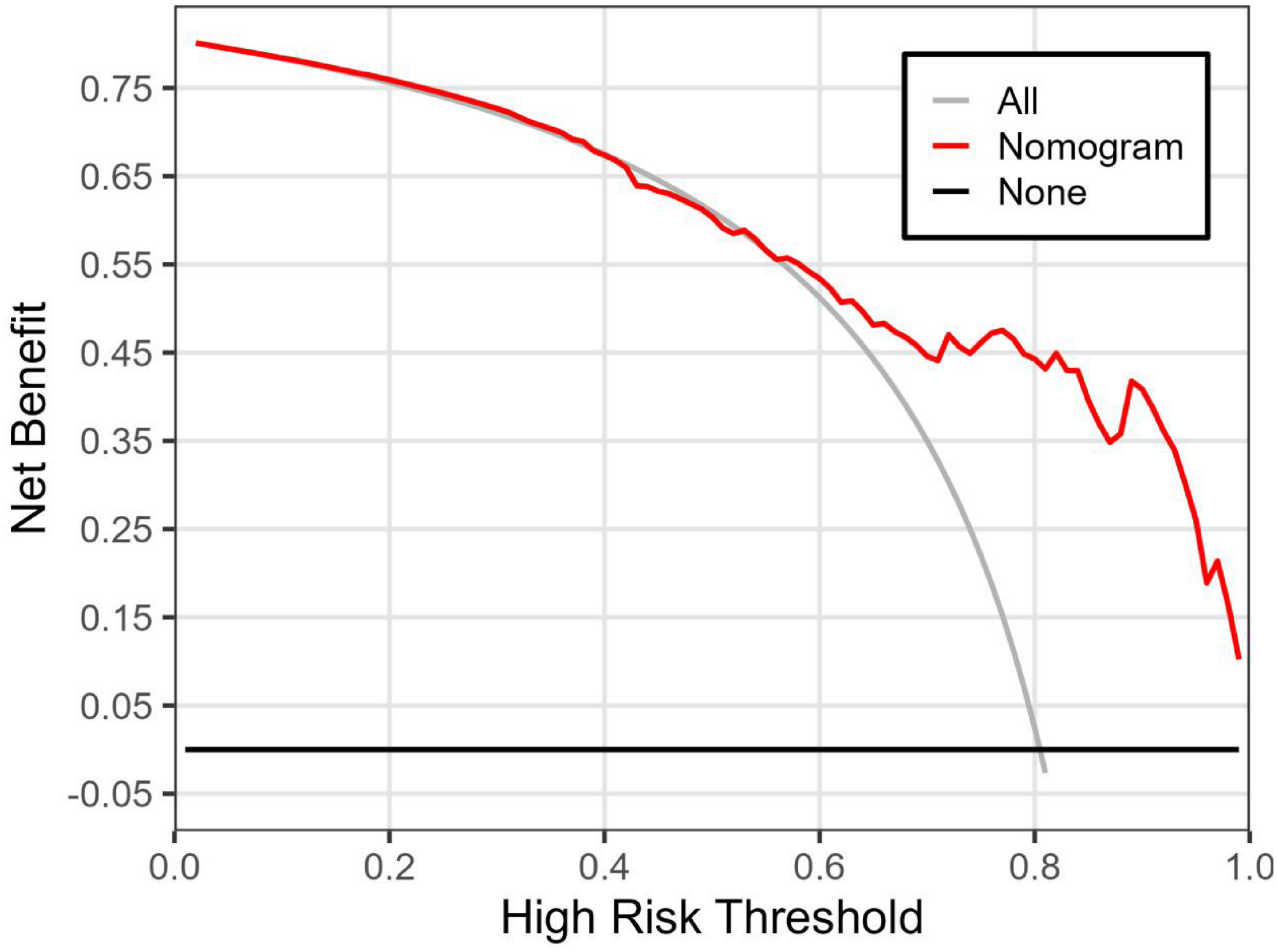
Decision curve analysis (DCA) of the frailty stratification model.

The Hosmer–Lemeshow goodness-of-fit test showed χ^2^ = 10.774, df = 8, and *P* = 0.215, while the Brier score was 0.125 ( < 0.250), indicating good calibration. The calibration curve (Figure 4) showed good agreement between the predicted probabilities and the observed probabilities, suggesting that the model had good calibration performance. Decision curve analysis (Figure 5) showed that the nomogram yielded a higher net benefit than the treat-all and treat-none strategies across a broad range of threshold probabilities, including clinically relevant higher thresholds between approximately 0.50 and 0.90. However, because frailty prevalence was high in this cohort and the analysis was based on internal data, this exploratory finding should be interpreted cautiously and should not be considered evidence that use of the nomogram improves clinical outcomes.

### 3.6 Sensitivity analysis excluding the Barthel Index

Because the Barthel Index measures functional independence and may clinically overlap with components of the Fried frailty phenotype, a sensitivity analysis was conducted by excluding the Barthel Index from the stratification model to examine whether model performance was mainly driven by this variable. After excluding the Barthel Index, the reduced model retained age, marital status, BMI, and number of involved vertebrae as predictors. The discrimination of the reduced model decreased slightly, with the AUC declining from 0.826 to 0.804 (Supplementary Figure S1). Bootstrap internal validation also showed a modest decrease in the optimism-corrected C-index, from 0.812 to 0.792. The calibration curve of the reduced model remained acceptable (Supplementary Figure S2), with a Hosmer–Lemeshow *P*-value of 0.183 and a Brier score of 0.132. Decision curve analysis showed a similar overall pattern, although the full model provided slightly higher net benefit at higher threshold probabilities (Supplementary Figure S3). Overall, the sensitivity analysis indicated that excluding the Barthel Index led to only a modest reduction in model performance, suggesting that the main findings were relatively robust.

### 3.7 Clinical threshold analysis

In clinical practice, different probability thresholds may serve different purposes. For frailty screening, the clinical priority is usually to identify as many potentially frail patients as possible and reduce missed cases, rather than to simply maximize the statistical balance between sensitivity and specificity. Therefore, the Youden-derived threshold may not fully align with the practical goal of early in-hospital frailty screening and risk stratification. Based on this consideration, we performed a clinical threshold analysis and selected three representative thresholds according to different clinical purposes: a lower screening threshold, the Youden-derived threshold, and a high-specificity threshold for resource prioritization. At the screening threshold of 0.600, sensitivity was 0.900 and specificity was 0.349. This threshold identified 234 frail patients and missed 26 frail patients. The PPV was 0.851, the NPV was 0.458, the LR + was 1.38, and the LR-was 0.286. This threshold may be more suitable for broad frailty screening because of its higher sensitivity and lower number of missed frail patients. At the Youden-derived threshold of 0.835, sensitivity was 0.665 and specificity was 0.905. The PPV was 0.966, the NPV was 0.396, the LR + was 6.99, and the LR- was 0.370. This threshold identified 173 frail patients but missed 87 frail patients. Therefore, although this threshold provided a better balance between sensitivity and specificity, it may be more suitable for identifying very high-risk patients than for broad frailty screening. At the high-specificity threshold of 0.890, specificity was 0.984 and PPV was 0.993. The LR + was 34.65. This threshold produced only one false-positive result but missed 117 frail patients. It may be useful for strict resource prioritization when clinical resources are limited, but it should not be used as a screening threshold. The selected threshold results are shown in Table 4.

**TABLE 4:** Clinical threshold performance of the full model.

Use	Cutoff	Sens	Spec	PPV	NPV	LR+	LR−	TP	FP	TN	FN
HS screening	0.600	0.900	0.349	0.851	0.458	1.38	0.286	234	41	22	26
Youden	0.835	0.665	0.905	0.966	0.396	6.99	0.370	173	6	57	87
High spec	0.890	0.550	0.984	0.993	0.346	34.65	0.457	143	1	62	117

HS, screening, high-sensitivity screening; High spec, high-specificity threshold; Sens, sensitivity; Spec, specificity; PPV, positive predictive value; NPV, negative predictive value; LR+, positive likelihood ratio; LR−, negative likelihood ratio; TP, true positives; FP, false positives; TN, true negatives; FN, false negatives.

## 4 Discussion

### 4.1 The high prevalence of frailty in older patients with spinal tuberculosis suggests that frailty screening should be moved forward to the early stage of hospitalization

In the present study, 260 of 323 older patients with spinal tuberculosis were classified as frail, yielding a frailty prevalence of 80.50%, indicating that this population is generally in a high-risk state characterized by reduced physiological reserve and impaired stress resilience. Frailty should not be regarded merely as a concomitant symptom; rather, it may run through the entire course of disease management and affect treatment tolerance and clinical prognosis. It therefore represents an important geriatric syndrome phenotype that warrants particular attention in this population. Although the primary model focused on identifying patients who met the Fried frailty threshold, pre-frailty should not be regarded as clinically unimportant. In the original Fried categories, 43 patients were classified as pre-frail, indicating that this subgroup still represented a clinically meaningful intermediate population. Pre-frail patients may benefit from earlier functional, nutritional, and rehabilitation interventions. Therefore, reporting the original Fried frailty categories separately helps preserve the clinical meaning of pre-frailty while maintaining the binary model for early identification of frailty. Compared with previous studies, the level of frailty observed in the present study was markedly higher. Li et al. reported a substantial frailty burden among older patients with pulmonary tuberculosis in China (49%) (14), but this level remained lower than that observed in our cohort of older patients with spinal tuberculosis. From a broader perspective, the prevalence of frailty has been reported to be approximately 10–11% in community-dwelling older adults (7, 22) and approximately 40–50% in hospitalized older adults (8, 23). These findings suggest that the frailty burden among hospitalized older patients with spinal tuberculosis is not only substantially higher than the baseline level in the general older population, but also higher than that in the overall hospitalized older population. Previous studies have shown that older patients with tuberculosis are often accompanied by chronic inflammation, nutritional depletion, and functional decline, which provides disease-specific background support for our findings (24–26). Therefore, by focusing on spinal tuberculosis as a specific subtype of tuberculosis, this study adds to the limited evidence regarding frailty in patients with osteoarticular and spinal involvement and provides more direct clinical evidence for early frailty screening and risk stratification in this population.

The high prevalence of frailty observed in this study may be jointly related to disease-specific burden, sample source, and measurement tools. Spinal tuberculosis is often accompanied by persistent inflammation and chronic consumptive status and commonly presents with back pain, structural vertebral destruction or deformity, and neurological impairment, all of which may further lead to activity limitation, prolonged bed rest, or deconditioning, thereby accelerating the decline in muscle strength and physical performance (27). At the same time, tuberculosis-related malnutrition and weight loss are common, and together with reduced physical activity, may promote a frailty progression pathway characterized by weight loss, decreased muscle strength, slow walking speed, and impaired activities of daily living (26). In addition, the study population consisted of hospitalized older patients with spinal tuberculosis, who generally had a relatively high disease burden, whereas the Fried frailty phenotype mainly focuses on physical and functional dimensions, including unintentional weight loss, weak grip strength, slow walking speed, low physical activity, and exhaustion (28). Patients with spinal tuberculosis often present with pain, activity limitation, and a consumptive state, and therefore may be more likely to be classified as frail under this assessment framework (27, 28). Therefore, the high prevalence of Fried frailty observed in this cohort should be interpreted cautiously in the clinical context of spinal tuberculosis. In this population, pain, vertebral instability, neurologic compromise, restricted ambulation, inflammation, and tuberculosis-related weight loss may directly contribute to Fried phenotype components. Accordingly, frailty identified in this study may reflect both baseline biological vulnerability and the acute or subacute burden of active spinal tuberculosis and disease-related immobility. Taken together, our findings suggest that older patients admitted with spinal tuberculosis may represent a frailty-enriched population requiring early frailty screening and multidimensional assessment, including functional status, nutritional risk, rehabilitation needs, pain control, social support, and discharge planning.

### 4.2 Analysis of the main factors associated with frailty in older patients with spinal tuberculosis

#### 4.2.1 Increasing age was associated with higher odds of frailty

The multivariable logistic regression analysis showed that age was an independent predictor associated with frailty in older patients with spinal tuberculosis. For each additional year of age, the odds of frailty increased (OR = 1.124, *P* < 0.001). This finding is consistent with previous evidence showing that frailty becomes more prevalent with advancing age, including among older adults with tuberculosis (6, 14). Age may reflect the progressive decline in physiological reserve across multiple systems, which increases vulnerability to frailty-related manifestations such as weakness, reduced mobility, and low physical activity (6, 29). In older patients with spinal tuberculosis, this age-related vulnerability may be further compounded by disease-related factors such as pain, vertebral destruction, neurological impairment, and prolonged activity limitation, all of which may accelerate functional decline (29–31). Therefore, the association observed in this study may reflect the combined effects of aging-related biological vulnerability and the additional burden imposed by spinal tuberculosis.

#### 4.2.2 Non-married status was significantly associated with frailty

The present study showed that marital status was significantly associated with frailty risk. Compared with married patients, non-married individuals (including those who were unmarried, divorced, or widowed) had significantly higher odds of frailty (OR = 6.078, *P* < 0.001). This finding suggests that, in addition to biomedical factors, social and family support may also play an important role in frailty among older patients with spinal tuberculosis. This result is generally consistent with previous evidence showing that non-married status is associated with higher odds of frailty in older adults (32). In hospitalized older patients with spinal tuberculosis, who often face substantial disease burden, pain, activity limitation, and nutritional depletion, the absence of stable family support may further compromise daily care, treatment adherence, and rehabilitation participation, thereby increasing vulnerability to frailty (14, 33). In addition, non-married status may also reflect a higher likelihood of social isolation and reduced emotional support, which have been linked to frailty progression in older adults (14, 34). Therefore, marital status may serve not only as a demographic characteristic, but also as an indirect indicator of social support availability in this population.

#### 4.2.3 Lower BMI was associated with higher odds of frailty and may indicate inadequate nutritional reserve

The multivariable analysis showed that BMI was significantly and negatively associated with frailty. For each 1 kg/m^2^ increase in BMI, the odds of frailty decreased (OR = 0.835, *P* = 0.002). In addition, BMI was significantly lower in the frail group than in the non-frail group (*P* < 0.05). These findings suggest that lower BMI may reflect inadequate nutritional and metabolic reserve and was associated with higher odds of frailty in older patients with spinal tuberculosis. This result is consistent with previous studies showing that low BMI is associated with frailty in older adults with tuberculosis (14), and more broadly with increased tuberculosis risk and disease burden (35). In the setting of spinal tuberculosis, where chronic consumption, pain, and activity limitation are common, lower BMI may also be accompanied by reduced muscle mass and poorer physical resilience, which may partly explain its association with frailty. Therefore, BMI may serve as a useful indicator for early frailty risk stratification at admission.

#### 4.2.4 An increased number of involved vertebrae reflected a greater disease burden and was associated with a higher probability of frailty

The multivariable logistic regression analysis showed that the number of involved vertebrae was positively associated with frailty. For each additional involved vertebra, the odds of frailty increased (OR = 1.278, *P* < 0.05). This finding suggests that the number of involved vertebrae may serve as an imaging marker reflecting greater disease burden in older patients with spinal tuberculosis. Previous studies have shown that the extent of vertebral involvement is associated with disease severity, structural destruction, and functional recovery in spinal tuberculosis (36–38). A greater number of involved vertebrae may indicate more extensive inflammation, pain, spinal instability, and activity limitation, all of which may increase vulnerability to frailty. In addition, patients with multivertebral involvement may experience more prolonged functional restriction and greater rehabilitation challenges, which may further contribute to frailty progression. Therefore, the number of involved vertebrae may provide useful information for early frailty risk stratification at admission.

#### 4.2.5 A lower Barthel Index was an important functional indicator associated with frailty

The multivariable logistic regression analysis showed that the Barthel Index was negatively associated with frailty. For each 1-point increase in the Barthel Index, the odds of frailty were lower (OR = 0.956, *P* < 0.001). This finding suggests that poorer functional status, particularly reduced independence in activities of daily living, was closely associated with frailty in older patients with spinal tuberculosis. The Barthel Index has long been widely used to assess functional independence and care needs (39), and lower admission Barthel Index scores in hospitalized older adults have been associated with worse clinical outcomes and greater vulnerability (40). In older patients with spinal tuberculosis, persistent pain, impaired spinal stability, neurological involvement, and reduced mobility may all contribute to ADL decline, reduced physical reserve, and increased dependence on caregiving, which may partly explain the observed association between lower Barthel Index scores and frailty (41, 42). Therefore, the Barthel Index may serve as a useful functional indicator for early frailty risk stratification at admission.

### 4.3 Performance of the screening nomogram and its interpretation for admission risk stratification

Frailty in older patients with spinal tuberculosis is jointly influenced by multiple factors, including demographic characteristics, nutritional reserve, functional status, and disease burden. Given the high prevalence of frailty and its substantial clinical consequences in this population, we used logistic regression to identify predictors associated with frailty and developed a nomogram-based stratification model. In the context of population aging and the continuing burden of tuberculosis, early frailty stratification may have relevance not only for individual risk recognition, but also for optimizing inpatient geriatric tuberculosis care pathways, multidisciplinary resource allocation, and timely supportive management. The results showed that the model had acceptable discrimination and calibration and may support admission risk stratification.

ROC analysis showed that the model had an AUC of 0.826 (95% CI: 0.779–0.873), indicating good ability to discriminate between frail and non-frail individuals. After bootstrap internal validation (*B* = 500), the original C-index of the model was 0.826 and the optimism-corrected C-index remained 0.812, suggesting that the model retained good discriminative ability and acceptable stability after internal validation. In terms of threshold interpretation, the Youden-derived cutoff of 0.835 showed high specificity but only moderate sensitivity. Therefore, this cutoff may be more appropriate for identifying patients at very high risk or for prioritizing limited supportive care resources, rather than for broad frailty screening. For screening purposes, the lower threshold of 0.600 may be more clinically appropriate because it achieved higher sensitivity and reduced the number of missed frail patients. These findings indicate that threshold selection should depend on the intended clinical use of the model, and the Youden-derived cutoff should not be interpreted as a universal clinical decision threshold. The model also showed good overall fit and calibration. The Hosmer–Lemeshow test yielded a *P*-value of 0.215, and the Brier score was 0.125, indicating good agreement between the predicted probabilities and the observed outcomes. Decision curve analysis further suggested that the model may provide a higher net benefit than both the treat-all and treat-none strategies across a broad range of threshold probabilities, including higher thresholds that may be more relevant for identifying very high-risk patients or prioritizing supportive care resources. This finding should be interpreted cautiously because frailty prevalence was high in the present cohort and the DCA was based on internally derived data. Therefore, the DCA results should be viewed as exploratory support for risk stratification rather than as definitive evidence of clinical utility or improved outcomes. By visualizing the model as a nomogram, clinicians and nurses may rapidly estimate an individual patient’s probability of frailty during the early stage of hospitalization based on key clinical characteristics, thereby facilitating early risk identification and in-hospital risk stratification. For patients identified as being at higher risk, the model may help inform closer assessment and earlier planning of supportive management, such as nutritional support, pain management, early rehabilitation training, and coordination of care resources. However, whether the use of this model can improve patient outcomes still requires further prospective and external validation.

From a clinical perspective, the model should be interpreted as supporting an admission risk-stratification pathway rather than providing a definitive prediction. For older patients with spinal tuberculosis who are identified as being at high risk of Fried frailty, the score may prompt further bedside multidimensional assessment, including geriatric assessment, nutritional evaluation, sarcopenia assessment, rehabilitation assessment, analgesia optimization, fall and pressure-injury prevention, delirium-risk review, medication reconciliation with attention to anti-tuberculosis drug toxicity, social support assessment, and discharge planning. This pathway-oriented interpretation is consistent with previous evidence from other acute-care populations suggesting that frailty screening can help trigger multidisciplinary care (43). However, whether this model-guided pathway improves clinical outcomes requires further prospective evaluation.

## 5 Limitations

This study still has several limitations. First, this was a single-center retrospective study conducted in a tertiary infectious disease specialty hospital, which may have introduced selection bias and limited the generalizability of the model. In addition, the outcome prevalence was high, with 260 patients classified as frail and 63 patients classified as pre-frail or non-frail, which may have affected the stability of coefficient estimation. Predictor selection was partly data-driven because univariate *P* < 0.05 was used as an initial screening criterion for preliminary candidate selection. Although the final model also considered clinical interpretability, measurement reliability, early in-hospital availability, and multicollinearity diagnostics, this approach may still exclude clinically relevant predictors and increase the risk of overfitting or overestimating the apparent effects of retained variables. Therefore, the final model should be interpreted cautiously, and future studies with larger and more balanced samples should consider prespecified clinically justified models, penalized regression, or shrinkage methods. Second, Fried frailty in this population may partly overlap with the clinical manifestations of active spinal tuberculosis. Spinal pain, vertebral instability, neurologic impairment, restricted ambulation, inflammation, weight loss, and functional limitation may directly influence Fried phenotype components, particularly walking speed and physical activity, and may therefore lead to possible misclassification of frailty status. In addition, functional predictors such as the Barthel Index may conceptually overlap with the Fried frailty phenotype, introducing possible circularity. Therefore, the model may partly capture current spinal tuberculosis disease burden and disease-related immobility, rather than baseline biological frailty alone. Third, this study did not directly assess sarcopenia or muscle quality. Although BMI, nutritional risk, grip strength, walking speed, and functional status were included in the assessment framework, objective measures of muscle mass or muscle quality, such as calf circumference, SARC-F, CT-based psoas muscle area, or skeletal muscle index, were not available in the retrospective dataset. In addition, the radiological component of the model was limited. Although several imaging-related variables were considered, more detailed parameters such as spinal canal compromise, neurological compression, and spinal deformity were not systematically included. Therefore, sarcopenia and more detailed radiological indicators should be incorporated into future prospective studies to improve the model’s value for multidisciplinary decision-making. Finally, the present study developed and internally validated a screening nomogram based on inpatient clinical data. The model has not yet undergone external validation, and no prospective outcome validation was performed. Although we propose that a high-risk score may help trigger a multidimensional assessment pathway, this pathway was not prospectively implemented or tested in the present study. Future multicenter prospective studies are needed to externally validate the model, further optimize its performance, and evaluate whether model-guided risk stratification can improve clinical outcomes.

## 6 Conclusion

In this single-center retrospective study, we developed a screening nomogram for identifying Fried frailty in older patients with spinal tuberculosis and performed bootstrap internal validation. The model, which incorporated age, marital status, BMI, number of involved vertebrae, and Barthel Index, showed acceptable discrimination and calibration in the present cohort. These findings suggest that the nomogram may serve as an auxiliary screening and admission risk-stratification tool to help identify patients who may benefit from further multidimensional assessment. The model should be interpreted within the context of its single-center retrospective design and internal validation. Further external validation and prospective impact studies are needed before this model can be recommended for broader clinical application.

## Data Availability

The original contributions presented in the study are included in the article/Supplementary material, further inquiries can be directed to the corresponding authors.
