# Supplementary material for: Development and internal validation of a frailty risk stratification model for older patients with spinal tuberculosis
Source: Front Med (Lausanne). 2026 Jul 21;13:1832932. doi: 10.3389/fmed.2026.1832932 (PMC13433300; doi:10.3389/fmed.2026.1832932)
Supplement: Supplementary file 1 [file Data_Sheet_1.docx]

Supplementary Figure S1. ROC curve comparison between the full model and the model excluding the Barthel Index.

Supplementary Figure S2. Calibration curve comparison between the full model and the model excluding the Barthel Index

Supplementary Figure S3. Decision curve analysis comparison between the full model and the model excluding the Barthel Index
